# Plasminogen Activation Inhibitor-1 Promotes Resilience to Acute Oxidative Stress in Cerebral Arteries from Females

**DOI:** 10.3390/ph17091210

**Published:** 2024-09-14

**Authors:** Safa, Charles E. Norton

**Affiliations:** Department of Medical Pharmacology and Physiology, University of Missouri, Columbia, MO 65212, USA

**Keywords:** calcium, endothelial cells, mitochondrial depolarization, reactive oxygen species, smooth muscle cells

## Abstract

Plasminogen activation inhibitor-1 (PAI-1) plays a central role in thrombus formation leading to stroke; however, the contributions of PAI-1 to cellular damage in response to reactive oxygen species which are elevated during reperfusion are unknown. Given that PAI-1 can limit apoptosis, we hypothesized that PAI increases the resilience of cerebral arteries to H_2_O_2_ (200 µM). Cell death, mitochondrial membrane potential, and mitochondrial ROS production were evaluated in pressurized mouse posterior cerebral arteries from males and females. The effects of pharmacological and genetic inhibition of PAI-1 signaling were evaluated with the inhibitor PAI-039 (10 µM) and PAI-1 knockout mice, respectively. During exposure to H_2_O_2_, PCAs from male mice lacking PAI-1 had reduced mitochondrial depolarization and smooth muscle cell death, and PAI-039 increased EC death. In contrast, mitochondrial depolarization and cell death were augmented in female PCAs. With no effect of PAI-1 inhibition on resting mitochondrial ROS production, vessels from female PAI-1 knockout mice had increased mitochondrial ROS generation during H_2_O_2_ exposure. During acute exposure to oxidative stress, protein ablation of PAI-1 enhances cell death in posterior cerebral arteries from females while limiting cell death in males. These findings provide important considerations for blood flow restoration during stroke treatment.

## 1. Introduction

Plasminogen activation inhibitor-1 (PAI-1) is a serine protease inhibitor that regulates the fibrinolytic system by inhibiting plasminogen activator. This decreases plasmin formation and fibrinolysis. Given these effects, PAI-1 is recognized as a key molecule in the pathogenesis and progression of thrombotic vascular events including stroke [1]. Consistently, elevated levels of PAI-1 can downregulate clot formation leading to infarction [2,3]. While clinical interventions for stroke are aimed at clot removal and the restoration of blood flow, the recanalization of ischemic tissue is often not achieved [4]. The reperfusion of the brain leads to damage to neurons and vascular cells [5]. Therefore, vascular damage may limit the ability of clot elimination to fully restore blood flow [6]. Understanding how to limit this damage has the potential to maintain blood flow control to the affected tissue and improve patient outcomes.

Reperfusion following ischemic stroke enhances the production of reactive oxygen species (ROS), which damage local tissue. In smooth muscle cells (SMCs) and endothelial cells (ECs), acute ROS exposure increases the intracellular Ca^2+^ concentration ([Ca^2+^]_i_) and causes apoptosis [7]. The depolarization of mitochondrial membrane potential (ΔΨ_m_) and an increase in mitochondrial ROS production are key events leading to apoptosis [7,8]. PAI-1 can regulate mitochondrial function [9] and limit apoptosis by inhibiting caspase 3 [10]. However, the ability of PAI-1 to modulate cell death in the cerebral vasculature in response to acute oxidative stress has not been defined.

Young women are protected against injury from ischemic stroke compared to men of the same age group [11,12]. In addition, SMCs from females are more resilient to cell death induced by H_2_O_2_ compared to males [13]. Given the protective actions of PAI-1, it may contribute to the greater resilience in cerebral arteries from females as PAI-1 expression is greater in large conduit vessels from females vs. males [14]. Therefore, we hypothesized that PAI-1 increases the resilience of posterior cerebral arteries (PCAs) of females to acute oxidative stress by limiting depolarization of ΔΨ_m_. Our findings reveal that genetic ablation, but not pharmacological inhibition, of PAI-1 promotes cellular resilience to ROS in females, but not males. These studies underscore the importance of sex differences when targeting the fibrinolytic pathway for stroke treatment.

## 2. Results

### 2.1. Genetic, but Not Pharmacological, Loss of PAI-1 Signaling Enhances H_2_O_2_-Induced Cell Death in Females 

**Figure 1:**
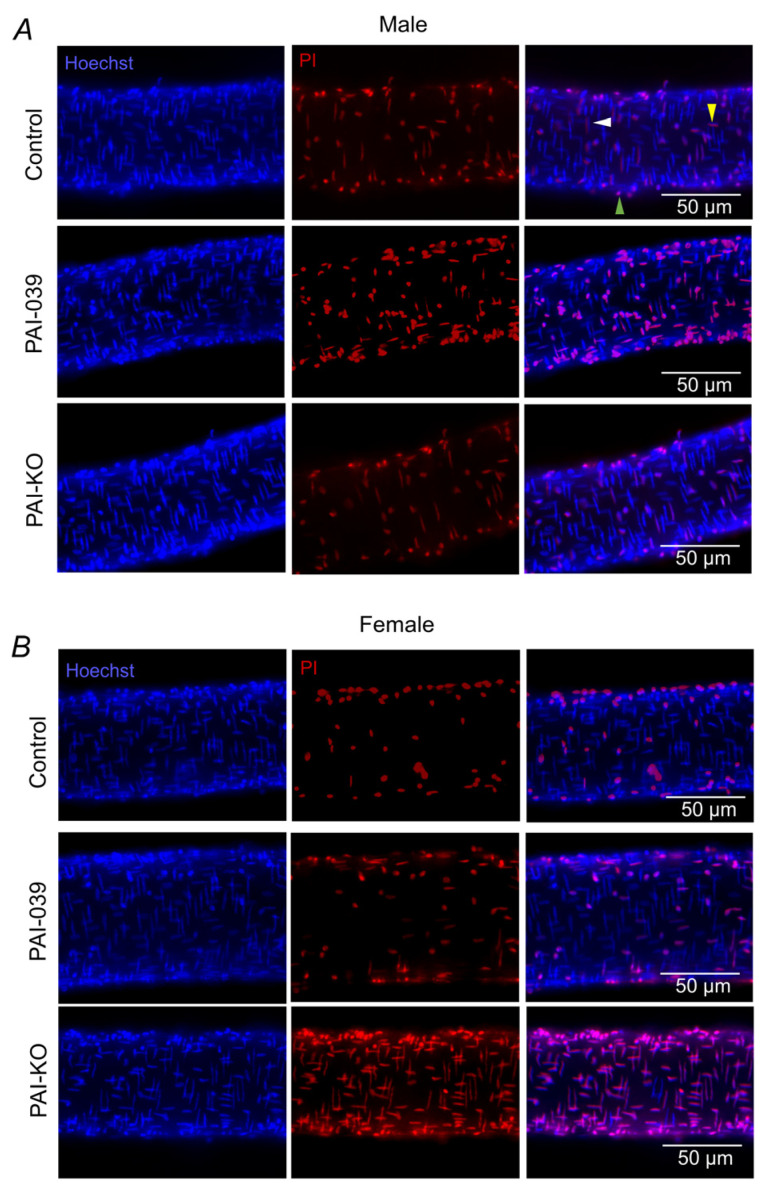
Effects of pharmacological inhibition and genetic ablation of PAI-1 on H_2_O_2_-induced cell death in PCAs from males and females. Representative images of Hoechst 33342 dye staining all the nuclei (left), propidium iodide (PI) staining the nuclei of dead cells (middle), and merged image (right) following 50 min exposure to H_2_O_2_ in isolated pressurized PCAs from male (**A**) and female (**B**) mice. In the upper right panel, the white arrow denotes a SMC nuclei, the yellow arrow denotes an EC nuclei, and the green arrow denotes an adventitial cell nuclei (not counted).

**Figure 2:**
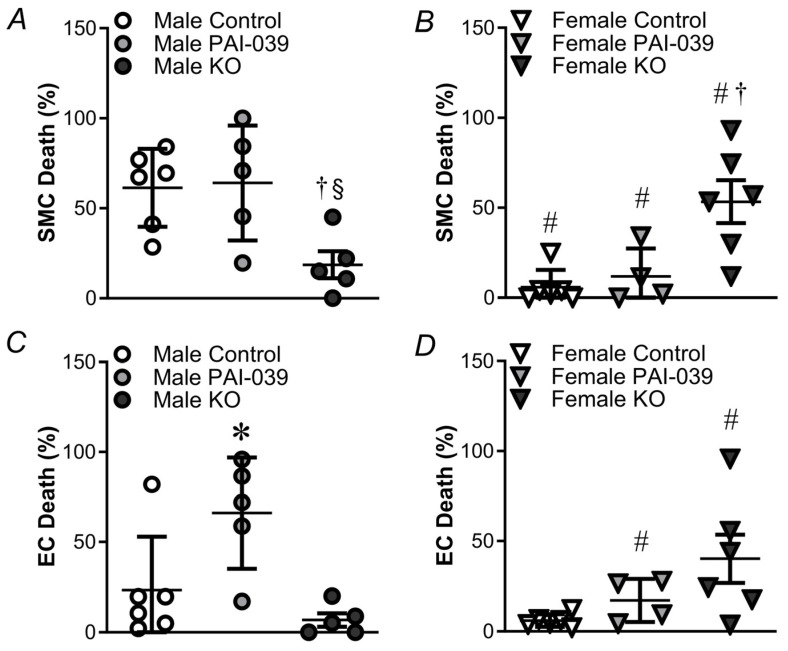
H_2_O_2_-induced cell death is greater in SMCs of females in the absence of PAI-1 protein expression. Male and female SMC (**A**,**B**) and EC (**C**,**D**) death following exposure to H_2_O_2_ for 50 min in the control/wildtype PCAs ± the inhibitor of PAI-1 (PAI-039 10 µM) or PAI-KO mice. Individual values with means ± SEM for n = 4–6/group. ^#^ *p* < 0.05, female vs. male, * *p* < 0.05, PAI-039 vs. control, ^†^ *p* < 0.05, PAI-KO vs. control. ^§^ *p* < 0.05, PAI-KO vs. PAI-039.

Consistent with previous findings [13], SMC death to H_2_O_2_ (200 µM, 50 min) was greater in PCAs from males vs. females (Figure 1 and Figure 2). The pharmacological inhibition of PAI-1 had a minimal effect on SMC death in males or females. In males lacking PAI-1 (PAI-KO), SMC death was attenuated (Figure 2A). In contrast, in females, PAI-KO led to a pronounced increase in SMC death (Figure 2B). EC death was minimal in both the male and female control mice (Figure 2C,D). The inhibition of PAI-1 increases EC death in males but not females. In contrast, for the PAI-KO mice, there was a significant increase in EC death in females vs. males. In the absence of H_2_O_2_, SMCs and ECs in PCAs have <1% death under these experimental conditions [7].

### 2.2. PAI-1 Levels in Brain and Cerebral Vasculature 

**Figure 3:**
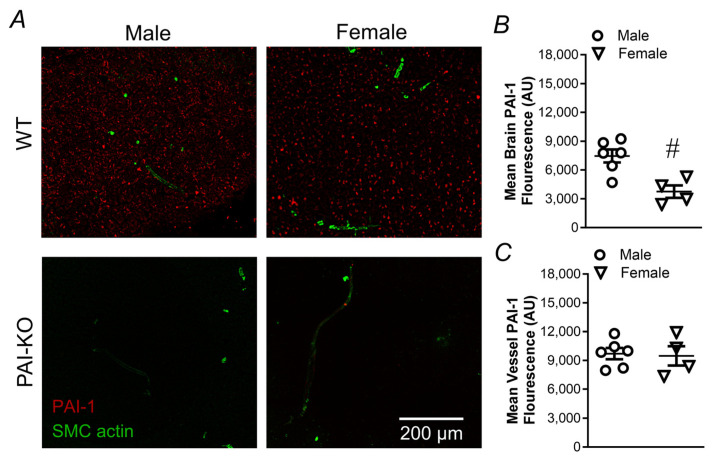
PAI-1 expression is greater in brain, but not vessels of males. (**A**) Representative images of PAI-1 (red) and SMC actin (green) from male and female wildtype and PAI-KO mice. Quantification of mean PAI-1 fluorescence in (**B**) whole brain slices and (**C**) regions containing blood vessels identified by SMC actin. Individual values with means ± SEM for n = 4–6/group. ^#^ *p* <0.05, female vs. male.

After observing a difference between sexes for responses to H_2_O_2_ during removal of PAI-1 signaling, we sought to evaluate how the PAI-1 levels differ in the brain. Total PAI-1 immunofluorescence in brain slices was greater in males vs. females (Figure 3B). In regions of the brain occupied by blood vessels (identified by SMC actin), PAI-1 fluorescence was significantly greater compared to nonvascular regions, yet similar for males and females (Figure 3C). The control experiments with PAI-KO mice verified minimal non-specific staining for PAI-1 (male: brain = 131 ± 59 AU, vessel = 231 ± 30 AU; female: brain = 287 ± 46 AU, vessel = 323 ± 123 AU; n = 2/group).

### 2.3. PAI-1 Attenuates the Sustained [Ca^2+^]_i_ Response Following H_2_O_2_ Exposure in Females

**Figure 4:**
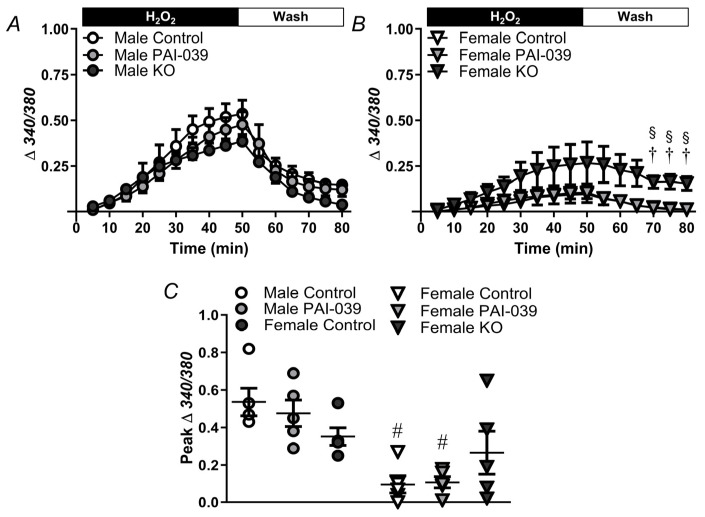
PAI-1 attenuates the sustained [Ca^2+^]_i_ response induced by H_2_O_2_ in females. [Ca^2+^]_i_ responses (changes in Fura-2 fluorescence) in PCAs obtained from males (**A**) and females (**B**) from wildtype/control mice in the absence and presence of PAI-039, and PAI-KO mice during 50 min exposure to H_2_O_2_, followed by a 30 min wash period. Note in females (**B**), PAI-039 data overlays control data. (**C**) The peak changes of [Ca^2+^]_i_ for both the male and female mice during the exposure to H_2_O_2._ Individual values and means ± SEM for n = 4–6/group. ^#^ *p* <0.05, female vs. male, ^†^ *p* < 0.05, PAI-KO vs. control. ^§^ *p* < 0.05, PAI-KO vs. PAI-039.

PCAs from control mice exhibited a significant increase in [Ca^2+^]_i_ in males (Figure 4A) upon exposure to H_2_O_2_, which returned to baseline levels after washout. Notably, the male control mice (Figure 4C) displayed a higher Ca^2+^ influx compared to females. However, in the male PAI-KO mice, no discernible differences were observed in the [Ca^2+^]_i_ levels compared to control mice. In the female PAI-KO mice exposed to H_2_O_2_, elevated calcium levels persisted compared to the controls (Figure 4B), and these levels did not return to baseline even after washing for 30 min in control PSS. PAI-039 had no significant effect in either males or females.

### 2.4. PAI-1 Limits ΔΨ_m_ Depolarization to H_2_O_2_ in Females

**Figure 5:**
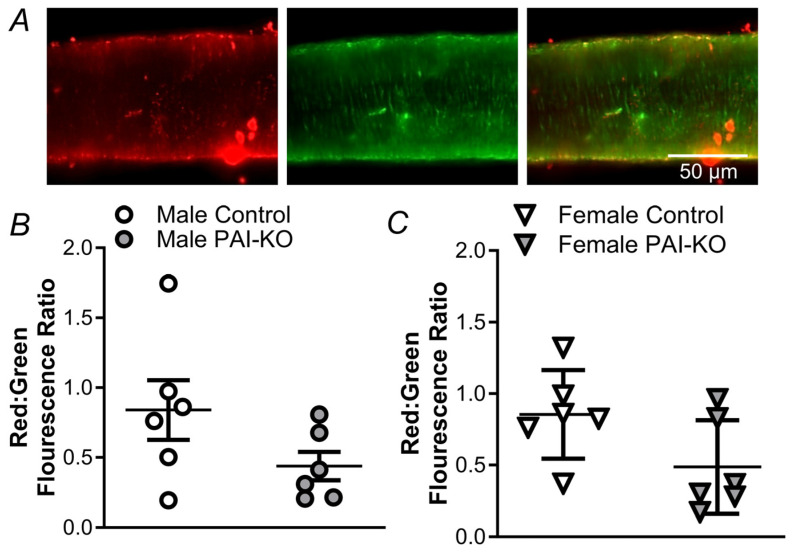
PAI-1 does not alter resting ΔΨ_m_. (**A**) Red (left) and green (center) JC-1 (5 µM) fluorescence as an index of the resting ΔΨ_m_ in an intact pressurized PCA from a male wildtype mouse by evaluating the red/green fluorescence ratio (right). The switch from green to red fluorescence results when the dye is dimerized at a high concentration within the mitochondria. The resting ΔΨ_m_ in the (**B**) male and (**C**) female PCAs from the control/wildtype and PAI-KO mice. Individual values with means ± SEM for n = 6/group. No significant differences were detected.

The intrinsic apoptosis pathway requires depolarization of ΔΨ_m_ [8], and a reduction in baseline ΔΨ_m_ could make it easier to reach the threshold required to trigger cell death. We utilized JC-1 to evaluate the resting ΔΨ_m_ in the presence and absence of PAI-1 expression. There was no difference in resting ΔΨ_m_ between the PCAs from the control male and female mice (Figure 5). In arteries from the PAI-KO mice, there was a trend for resting ΔΨ_m_ to be lower compared to the control mice in males (*p* = 0.12) and females (*p* = 0.07).

**Figure 6:**
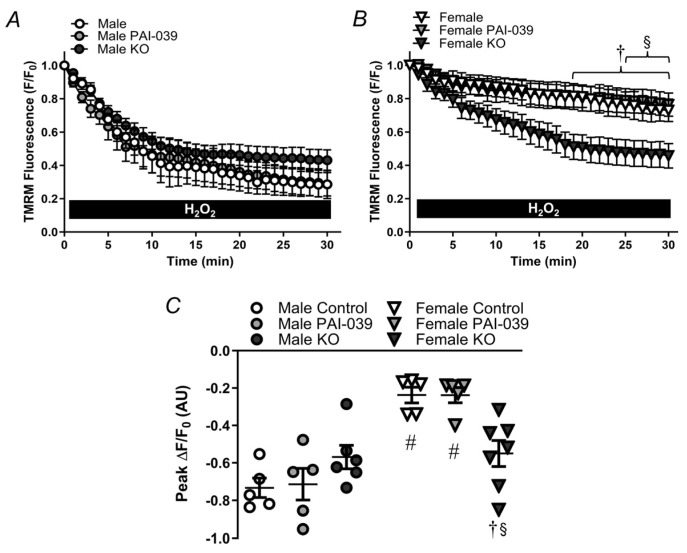
Genetic deletion of PAI-1 enhances depolarization of ΔΨ_m_ to a greater extent in females than males. (**A**) Changes in ΔΨ_m_ (TMRM fluorescence; 10 nM; F/F_0_) during H_2_O_2_ (200 µM) exposure in PCAs from control male and PAI-KO male mice. (**B**) Changes in ΔΨ_m_ during H_2_O_2_ exposure in PCAs from control female and PAI-KO female mice. (**C**) Peak changes in ΔΨ_m_ during H_2_O_2_ exposure in PCAs from males and females (control, PAI-039, PAI-KO). Individual values and means ± SEM for n = 5–7/group. ^#^ *p* < 0.05, female vs. male, ^†^ *p* < 0.05, PAI-KO vs. control. ^§^ *p* < 0.05, PAI-KO vs. PAI-039.

We utilized TMRM to monitor alterations in ΔΨ_m_ in the presence of H_2_O_2_ in both the control and PAI-KO mice. The ΔΨ_m_ depolarization in the presence of H_2_O_2_ shows a progressive decline of ΔΨ_m_ with the magnitude of depolarization greater in males compared to females in the wildtype mice in the presence and absence of PAI-039 (Figure 6). However, similar to cell death (Figure 2), PCAs from the female PAI-KO mice exhibited a greater decline in ΔΨ_m_ during exposure to H_2_O_2_ compared to their wildtype counterparts.

### 2.5. PAI-1 Limits Mitochondrial O_2_^−^ Production during Acute Oxidative Stress in Females

**Figure 7:**
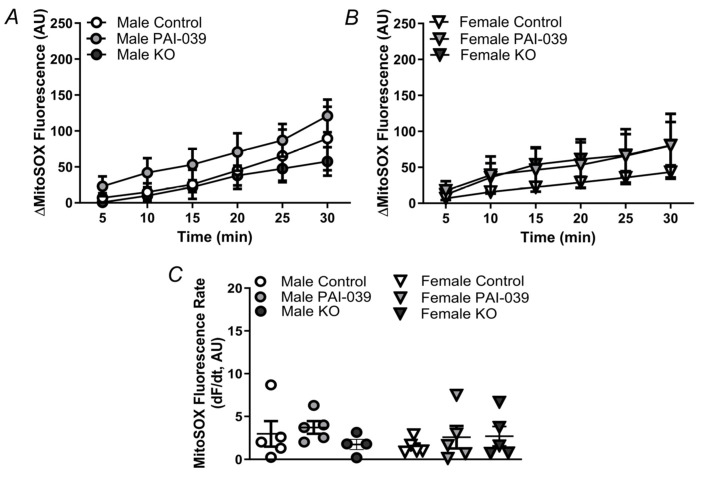
PAI-1 Inhibition does not alter mitochondrial ROS production under baseline conditions. MitoSOX fluorescence accumulation under baseline conditions in male (**A**) and female (**B**) control/wildtype, PAI-039-treated, and PAI-KO PCAs. (**C**) MitoSOX fluorescence rate for PCAs from males and females for each treatment. Individual values and means ± SEM for n = 5–6/group. No significant differences were detected.

**Figure 8:**
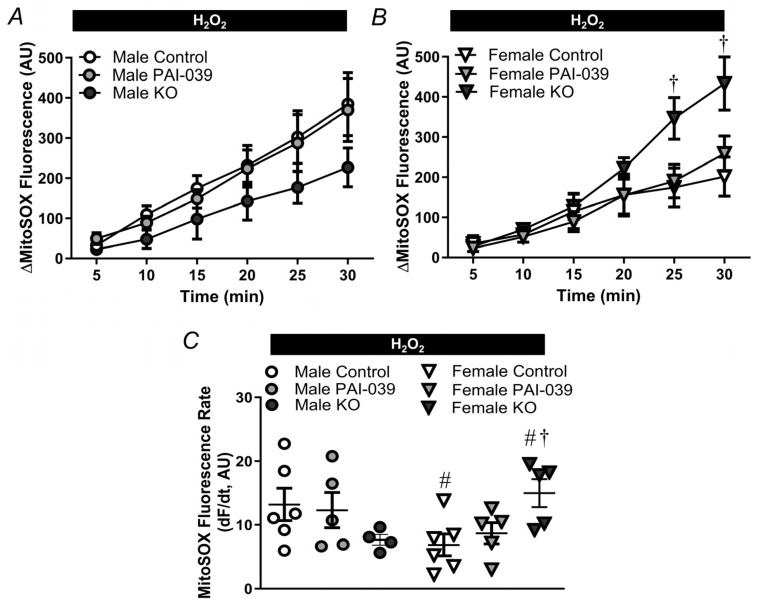
Genetic deletion of PAI-1 enhances mitochondrial ROS production in females during acute oxidative stress. MitoSOX fluorescence accumulation under baseline conditions in male (**A**) and female (**B**) control/wildtype, PAI-039-treated, and PAI-KO PCAs. (**C**) MitoSOX fluorescence rate for PCAs from males and females for each treatment. Individual values and means ± SEM for n = 4–6/group. ^#^ *p* < 0.05, female vs. male, ^†^ *p* < 0.05, PAI-KO vs. Control.

Under resting conditions, PAI-1 does not alter mitochondrial O_2_^−^ production in either males or females (Figure 7) and ROS production was not different between sexes. During H_2_O_2_ exposure, mitochondrial O_2_^−^ production was enhanced in both males and females, with significantly greater O_2_^−^ generation in males vs. females for the control and PAI-039 groups (Figure 8). The vessels from female PAI-KO mice had increased mitochondrial ROS production compared to PCAs from the wildtype mice with O_2_^−^ production similar to males. 

## 3. Discussion

Damage from ROS is integral to the negative outcomes associated with reperfusion during the treatment of stroke. Presently, we assessed the role of PAI-1 in mediating protection against ΔΨ_m_ depolarization and cell death induced by ROS in cerebral arteries from males and females using the pharmaceutical inhibition and genetic deletion of PAI-1. Our key findings are that in response to acute oxidative stress (200 μM, 50 min), cerebral vessels from the female PAI-KO mice are more susceptible to ΔΨ_m_ depolarization and cell death, the loss of PAI-1 increases resilience to cell death in males without altering the ΔΨ_m_ response, and PAI-1 limits mitochondrial O_2_^−^ production in females. Collectively, our data indicate a novel role for PAI-1 in regulating the initiation of apoptosis in cerebral arteries exposed to oxidative stress.

### 3.1. PAI-1 Enhances Resilience to Oxidative Stress in Cerebral Arteries from Females

Stroke is a sexually dimorphic disease with men having higher rates of stroke compared to females [11,12]. Men also have poorer functional outcomes, higher mortality rates, and a higher risk for recurrent vascular events compared to women [15,16]. We have previously shown that PCAs from females are more resilient to cell death induced by H_2_O_2_ than males [13]. Females have multiple mechanisms including estrogen and the X-linked inhibitor of apoptosis (XIAP) that can reduce apoptosis by limiting the activation of caspases [17,18]. However, these effects occur after the initiation of apoptosis in the mitochondria. As the structure and function of mitochondria differ between sexes [19], it is important to identify how females may also limit the initiation of apoptosis in this organelle.

PAI-1, recognized for its thrombotic properties, can also play a role in cellular survival. In isolated pressurized PCAs, our findings illustrate that vessels from female PAI-KO mice are more susceptible to damage resulting from H_2_O_2_ exposure compared to their wildtype counterparts or the mice treated with PAI-039 (Figure 2). These findings contrast with their male counterparts, which are more resilient to H_2_O_2_-induced cell death in the absence of PAI-1 expression. These findings point to distinct differences evoked by pharmacological inhibition and protein ablation of PAI-1 and illustrate a remarkable difference in the role of PAI-1 between sexes. Our findings are consistent with those in cultured vascular SMCs where the overexpression of PAI-1 limits apoptosis induced by tumor necrosis factor α by a direct interaction of PAI-1 with caspase 3 inhibiting its activity [20]. Additionally, PAI-1 has an anti-apoptotic role in neurons [21], and heightened levels of PAI-1 in breast cancer contribute to malignant cell survival [22]. On the other hand, in Chinese hamster ovary cells, PAI-1 overexpression increases apoptosis [23]. These differences suggest that PAI-1’s ability to modulate survival may differ across tissues or stimuli used to evoke cell death. 

To determine whether sex differences in vascular death to H_2_O_2_ are mediated by differences in PAI-1 expression, we labeled brain sections containing neurons, astrocytes, blood vessels, and capillaries for PAI-1. In the regions occupied by blood vessels, we detected no differences in PAI-1 fluorescence. However, other reports indicate that PAI-1 expression in the aorta is higher in females than males [14], so such effects may also be dependent upon vessel size or location. In addition to vascular cells, astrocytes and neurons also produce PAI-1 [24]. PAI-1 levels were greater in whole brain sections of males compared to females. PAI-1 expression was also greater in the vascular vs. nonvascular regions of the brain, consistent with previous reports [25]. While our studies focus specifically on vascular PAI-1, the principal location for PAI-1 is in the plasma, and men have higher circulating PAI-1 levels compared to women [26]. These differences in the respective levels of PAI-1 across various tissue compartments may have important implications for the modulation of cellular responses.

Increases in [Ca^2+^]_i_ are vital for cell death to H_2_O_2_ in PCAs [7]. Therefore, we determined whether PAI-1 mediates differences in the Ca^2+^ response to oxidative stress. For all treatment groups, H_2_O_2_ evoked a greater increase in males vs. females (Figure 4). While PAI-1 inhibition/knockout did not significantly alter the increase in [Ca^2+^]_i_ during H_2_O_2_ exposure, the vessels from female PAI-KO mice had a sustained increase in [Ca^2+^]_i_. This remaining Ca^2+^ following the withdrawal of H_2_O_2_ is likely mediated by cell death pathways. The activation of caspases 3 and 9 elevates [Ca^2+^]_i_ [27], and these caspases play a critical role in vascular cell death to H_2_O_2_ [28]. Nonetheless, it does not appear that PAI-1 modulates the initial cytosolic Ca^2+^ response which triggers cell death within the mitochondria. 

### 3.2. PAI-1 Limits Mitochondrial Disruption during Acute Oxidative Stress

Although PAI-1 has roles in mitochondrial structure and function, the role of PAI-1 in regulating mitochondrial dysfunction is poorly defined. We observe a trend for lower (depolarized) resting ΔΨ_m_ in PCAs from both males and females of PAI-KO mice (Figure 5). PAI-1 may reduce ΔΨ_m_ by increasing mitochondrial uncoupling protein (UCP) expression. Located on the inner mitochondrial membrane, UCPs allow for the re-entry of protons into the matrix, thereby uncoupling respiration from protein synthesis, and can play an important role in redox signaling [29]. In skeletal muscle, PAI-1 deficient mice have an upregulation of UCP 3 [30], and PAI-1 inhibition enhances the expression of UCP 1 [31]. While this has not been addressed in vascular cells, such changes would enhance proton leak dissipating ΔΨ_m_ and make it easier to reach the threshold mitochondrial depolarization required to initiate apoptosis. Furthermore, UCPs 2 and 3 can be activated by ROS [32] which may exacerbate the depolarization of ΔΨ_m_ during conditions of acute oxidative stress.

Consistent with the previous findings from our laboratory [13], we observed that H_2_O_2_ evoked a progressive loss of ΔΨ_m_ with a more robust effect in PCAs from males vs. females. Similar to cell death (Figure 2), the depolarization of ΔΨ_m_ was not altered by the PAI-1 inhibitor in males or females (Figure 6). However, protein ablation of PAI-1 in female PCAs made them more vulnerable to H_2_O_2_-induced depolarization compared to males. While the reason for this phenomenon requires further investigation, PAI-1 can increase mitochondrial fragmentation in cancer cells [9], and mitochondrial fission reduces the susceptibility to oxidative stress-induced cell death [33]. In addition, mitochondrial fission reduces apoptosis mediated by Ca^2+^ overload in HeLa cells [34]. Such structural changes would require time to take effect and may relate to why reduced cell death is observed in the vessels from female PAI-KO mice but not from the PCAs treated with the PAI-1 inhibitor. An alternative explanation for our findings is that PAI-1 promotes a shift to glycolytic metabolism [9] and increased reliance on glycolysis promotes resilience to apoptosis [35]. Whether PAI-1 evokes these changes in mitochondrial structure and metabolism in the context of acute oxidative stress in cerebral arteries merits further study. 

The production of mitochondrial ROS is key for the initiation of apoptosis [36]. We have previously shown that mitochondrial O_2_^−^ production is enhanced by H_2_O_2_ exposure and contributes to cell death induced by H_2_O_2_ in PCAs [7]. Consistently, PCAs from each group had elevated mitochondrial ROS generation during exposure to H_2_O_2_ (compare Figure 8 to Figure 7). Furthermore, the magnitude of mitochondrial O_2_^−^ generation during H_2_O_2_ exposure followed the pattern for SMC death observed in Figure 1 and Figure 2. The propagation of ROS signals in this regenerative phenomenon can enhance vascular permeability and augment the spread of the ischemic penumbra during stroke [37,38].

### 3.3. Differences in Pharmacological and Genetic Targeting of PAI-1 and Other Limitations

A key remaining question relates to the differing effects of pharmacological inhibition and protein ablation of PAI-1 to limit depolarization of ΔΨ_m_ and vascular apoptosis. Whereas genetic deletion depletes the protein completely, the inhibitor blocks enzymatic activity leaving the other functions, such as protein/protein interactions, intact. Notably, the inhibitor utilized in these studies, PAI-039, does not inhibit PAI-1 when it is bound to vitronectin [39]. Vitronectin is a glycoprotein excreted by the liver which binds to PAI-1 stabilizing it [40]. Therefore, potential interactions of PAI-1 with vitronectin may limit the ability of PAI-039 to modulate cell death in this setting. Furthermore, whether there are sex differences in vitronectin levels or interactions with PAI-1 remains to be investigated. Differences between the pharmacological and genetic inhibition of PAI-1 may also reflect changes in gene expression due to the transcriptional actions of PAI-1 [41]. Although whether cellular resilience in females results from changes in gene expression resulting from PAI-1 deletion remains to be established, it is possible that such changes require a greater timeline to take effect than for our present studies acutely pretreating arteries with the PAI-1 inhibitor.

An additional complicating factor for the interpretation of our results is that PAI-1 can inhibit caspase 3 [20]. While this action may further limit the cell death associated with exposure to H_2_O_2_, our findings establish a novel role for PAI-1 to limit the depolarization of ΔΨ_m_ to acute oxidative stress. Nonetheless, some contribution of caspase inhibition by PAI-1 to reduce cell death in females cannot be ruled out. Another limitation is the small area occupied by blood vessels in each brain section which may have reduced our ability to detect subtle differences in PAI-1 levels in vascular cells.

### 3.4. PAI-1 in Stroke

Thrombolytic treatment for ischemic stroke with intravenous tissue plasminogen activator is a beneficial treatment for stroke despite the increased risk for subsequent intracerebral hemorrhage [42]. Furthermore, patients with cerebral vessel occlusion treated with thrombectomy and tissue plasminogen activator often have incomplete reperfusion [43]. Ameliorating the adverse outcomes associated with these treatments has the potential to improve patient outcomes. Herein, we identify the potential for PAI-1 to limit the depolarization of ΔΨ_m_ and concomitant apoptosis to acute oxidative stress, which occurs during reperfusion following clot removal. We have previously shown that limiting apoptosis preserves vascular reactivity [7] which would be expected to enhance reperfusion and blood flow control in the setting of stroke. This is supported by findings in PAI-1 overexpressing mice, where following focal ischemia resulting from mechanical stimulation, there is reduced infarct size compared to the wildtype controls [1]. Furthermore, PAI-1 can limit plasminogen activator-induced blood/brain barrier leakage [44] and directly enhance endothelial tight junction properties [45] to limit cerebral edema. At present, how plasminogen activator therapy may alter vascular function in the setting of ischemia/reperfusion is poorly understood. By understanding sex differences in the role of PAI-1 in mitochondrial apoptosis and vascular survival, treatments can target PAI-1’s beneficial effects, maintaining vascular viability and the restoration of perfusion to the affected tissue following stroke.

PAI-1 may also play a role in the increased risk for stroke observed during advanced age and obesity [46,47]. PAI-1 levels are increased in the settings of advanced age [26] and obesity [48]. However, despite the increase in stroke incidence, both aging and high fat, Western-style diet protect the vasculature from acute oxidative stress [13,49]. However, whether PAI-1 contributes to this protection requires experimentation in aged and Western diet mice lacking the PAI-1 gene to substantiate this interpretation. In contrast, PAI-1 may also have detrimental effects. The inhibition of PAI-1 improves collateral perfusion and reduces infarct size in leptomeningeal anastomotic arterioles of aged hypertensive rats [50]. Future investigations are required to determine how to best modulate levels of PAI-1 to optimize stroke outcomes in these settings. Similar consideration should be given to myocardial infarction where elevated levels of PAI-1 precede heart attack [51] and remain elevated in survivors [52].

### 3.5. Conclusions

PAI-1 has been implicated in a wide range of cardiovascular diseases including atherosclerosis, coronary artery disease, venous thrombosis, myocardial infarction, and stroke [53]. However, PAI-1’s function in modulating vascular cell death during acute oxidative stress has not been defined. Our findings highlight distinct sex differences in the role of PAI-1 in promoting cellular resilience to oxidative stress in cerebral arteries from females with a potentially detrimental effect in males. These complex interactions should be considered along with the primary role of PAI-1 to regulate thrombus formation when using clot busters for the treatment of acute oxidative injuries such as ischemic stroke and myocardial infarction.

## 4. Materials and Methods

### 4.1. Animal Care and Use

The experimental procedures were reviewed and approved by the University of Missouri Animal Care and Use Committee and comply with the guidelines for this journal. Male and female young (Bar Harbor, ME, USA; 3–6 months) C57BL/6J and C57BL/6J-congenic PAI-1 (Serpine1)-deficient (*Serpine1*^−/−^) (PAI-KO) mice were provided by Dr. William Fay, University of Missouri, Columbia. All mice were housed on a 12:12 h light/dark cycle at ~23 °C with fresh water and food available ad libitum. The mice were anesthetized with ketamine and xylazine (100 mg·kg^−1^ and 10 mg·kg^−1^, respectively; intraperitoneal injection) to harvest the brain tissue and killed by decapitation.

### 4.2. Preparation of Isolated Posterior Cerebral Arteries

The mouse brain was removed from the skull and affixed onto a silicone rubber surface, and then immersed in a chilled physiological salt solution [PSS, pH 7.4; in mM: 140 NaCl (Thermo Fisher Scientific, Waltham, MA, USA), 5 KCl (Fisher Scientific), 1 MgCl_2_ (Sigma-Aldrich, St. Louis, MO, USA), 10 HEPES (Sigma), and 10 glucose (Fisher)]. An unbranched segment (~2 mm) of the PCA was dissected by carefully removing the surrounding tissues and then cannulated onto a fire-polished micropipette and secured in place using a silk suture. Each vessel was superfused with PSS at room temperature, pressurized to 90 cm H_2_O (~70 mmHg), and maintained at a constant temperature of 37 °C throughout each experiment.

### 4.3. Quantification of Cell Death

Vessel cannulas were pre-loaded with a mixture of PSS; propidium iodide (PI; 2 µM; Cat. no. P4170, Sigma)—a dye staining dead and dying cell nuclei; and Hoechst 33342 (1 µM; Cat. no. H1399, Fisher)—a dye that stains all cell nuclei (Figure 1). The cannulated and pressurized vessels were exposed to H_2_O_2_ (200 µM) at a concentration consistent with cerebral ischemia/reperfusion injury in rat [54] via superfusion at a rate of 3 mL/min for 50 min. To evaluate the effects of PAI-1 inhibition, PCAs were pretreated with PAI-039 (10 µM; Cat. no. 393105-53-8, Sigma-Aldrich) [55] for 20 min and PAI-039 was included in the PSS during the 50 min H_2_O_2_ exposure. This pharmacological treatment regimen has been validated as an effective protocol to evaluate a variety of pharmacological inhibitors on cell death [7,13,28,49]. To evaluate how the loss of PAI-1 protein expression affects cell death, experiments were repeated in PAI-KO mice. Following a 50 min exposure to H_2_O_2_, normal superfusion with standard PSS was reinstated, and the vessel lumen was perfused with PSS with the nuclear dyes. Image stacks were acquired from the upper half of each vessel using a 40× water immersion objective coupled to a DS-Qi2 camera with Elements software (version 4.51) on an E800 microscope (all from Nikon, Tokyo, Japan). Appropriate filters for imaging the fluorescent dyes Hoechst 33342 and PI were used. The EC nuclei aligned parallel to the vessel axis and the SMC nuclei oriented perpendicular to the vessel axis were counted within a defined region of interest (100 × 300 µm; containing ~50 nuclei of each cell type) for both dyes. Subsequently, the number of total and dead cells was analyzed in Image J (NIH, version 1.51k; Bethesda, MD, USA) [7,28]. Cell death was quantified as (# nuclei stained with PI/# nuclei stained with Hoechst 33342) × 100%. 

### 4.4. Immunofluorescence for PAI-1 Expression 

Coronal sections (thickness, 1 mm) were cut from the excised brains in the approximate perfusion field of PCAs, placed into optimal cutting temperature compound (OCT; Cat no. 23-730-571, Fisher), frozen in 2-methylbutane (Cat no. 60-48-071, Fisher), placed in liquid nitrogen, and stored at −80 °C [13]. The tissues were sectioned (thickness, 16 µm) using a cryostat (HM550, Fisher), maintained at 18 °C, and placed onto Superfrost slides (Cat no. 1255015, Fisher). Each section was then circled by a PAP pen and washed with phosphate-buffered saline (PBS, pH 7.4; Cat no. P3813, Sigma). The sections were permeabilized for 15 min with 0.5% Triton X-100 (Cat no. T8787, Sigma) in PBS, rinsed, and then blocked for 15 min with 10% normal goat serum (Cat no. 566380, Sigma) in PBS. The brain coronal sections were then incubated with a rabbit polyclonal antibody for PAI-1 (1:250; Cat no. ab66705, Abcam, Cambridge, UK) [56] for 1 h. The preparations were then washed twice with PBS and then incubated with an Alexa Fluor 647 conjugated donkey anti rabbit secondary antibody (1:250; Cat no. A-31573, Fisher) and a Dylight 488 conjugated antibody for SMC actin (1:500; Cat no. NBP2-47699G, Novus Biologicals, Centennial, CO, USA) for 1 h. The slides were rinsed 3 times with PBS, and Prolong Diamond (Cat no. P36965, Fisher) was added before the sections were sealed with a coverslip. 

To visualize fluorescence, slides were placed on the stage of a Leica (Wetzlar, Germany) DMi8 microscope and illuminated with an LED light source. Images were acquired with an HC PL APO 40×/0.95 objective using a K8 monochrome camera and THUNDER imaging technology (LASX software, version 3.9.1.28433) using consistent settings for all image acquisitions. The mean fluorescence intensity for PAI-1 was quantified for each image using Image J. In addition, the PAI-1 fluorescence intensity was selectively evaluated in regions of interest set to match the area occupied by the blood vessels identified by SMC actin. For each sample, three 900 × 1500 µm regions of the brain were captured and averaged for a single value. The mean PAI-1 fluorescence intensity was compared between male and female brains from the wildtype mice, with the brains from the PAI-KO mice serving as a negative control.

### 4.5. Calcium Photometry

Isolated, pressurized PCAs were positioned on the stage of the Nikon Eclipse TS100 inverted microscope and incubated with Fura 2-am dye (dissolved in DMSO and diluted to a concentration of 1 µM in PSS, resulting in a final [DMSO] of 0.5%; Cat. No. F14158, Fisher Scientific) for 40 min in a static bath. To remove excess dye, the vessels were superfused with PSS for 20 min. [Ca^2+^]_i_ was assessed using Fura-2 fluorescence. The preparation was alternately excited at 340 and 380 nm, while emissions at 510 nm were recorded using a 20× Fluor 20 Nikon objective (NA = 0.45). IonWizard 6.3 software (IonOptix) was utilized for recording [Ca^2+^]_i_ [7,28]. After baseline fluorescence was established, 200 µM H_2_O_2_ was introduced into the superfused solution. Calcium signals were recorded in the vessels from wildtype control mice in the presence or absence of PAI-039 and PAI-KO mice at a rate of 10 Hz for 30 s at 5 min intervals (to reduce Fura-2 dye photobleaching) during a 50 min exposure to H_2_O_2_, followed by a subsequent 30 min wash with control PSS.

### 4.6. Mitochondrial Membrane Potential

Pressurized PCAs from wildtype and PAI-1 knockout mice were loaded in a static bath with 2 mL of JC-1 (5 µM; Cat. No. T3168, Fisher), a ratiometric ΔΨ_m_-dependent dye, for 20 min. JC-1 accumulates within the mitochondria as it is negatively charged and forms dimers that fluoresce red, whereas the monomers fluoresce green [57]. Thus, more hyperpolarized mitochondria would have a greater red/green fluorescence ratio. Images were captured with appropriate filters and fluorescence ratios (red/green) were quantified within a region of interest (100 × 300 µm). The ratio of background-subtracted red/green fluorescence was used as an index for the resting/baseline ΔΨ_m_.

To evaluate how H_2_O_2_ changes ΔΨ_m_, PCAs were loaded with TMRM (10 nM; Cat. no. 115532-50-8; Sigma-Aldrich), a fluorescent indicator of mitochondrial ΔΨ_m_, via the bath for 30 min without superfusion [13,49]. Images of PCAs were acquired with an Olympus MVX10 microscope (Tokyo, Japan) with an MV PLAPO 2× objective (NA = 0.5, Olympus) coupled to a megapixel CCD camera (XR/Mega10, Stanford Photonics, Palo Alto, CA, USA) at a final magnification of ~120× with the excitation of TMRM at 543/22 nm and emissions recorded at 592/40 nm. After establishing baseline fluorescence in arbitrary units (AUs), measurements were acquired every minute for 30 min of H_2_O_2_ (200 µM) exposure. The responses were evaluated in vessels from wildtype control mice in the presence or absence of PAI-039 and PAI-KO mice. Previous experiments with the protonophore FCCP (10 µM) have verified the TMRM as an effective tool to measure changes in ΔΨ_m_ in the vessel wall [13].

### 4.7. Quantification of Mitochondrial ROS Production

To assess the specific generation of mitochondrial O_2_^−^, MitoSOX™ (5 µM; Cat. no. M36008, Fisher) was introduced to PCAs from wildtype control mice in the presence or absence of PAI-039 and PAI-KO mice in a static bath. Subsequently, O_2_^−^ levels were evaluated at one-minute intervals for a duration of 30 min. To investigate the impact of H_2_O_2_ on mitochondrial superoxide production, the PCAs were treated with H_2_O_2_ (200 µM). We have previously demonstrated the selectivity of MitoSOX in this preparation using positive and negative controls [7]. Linear regression was used to determine the rate of increases in MitoSOX fluorescence.

### 4.8. Statistics

The data were analyzed using Student’s unpaired *t*-tests or analysis of variance (Prism 9, GraphPad Software; La Jolla, CA, USA) as appropriate. When significant main effects were detected with Two-way ANOVA, post hoc comparisons were performed using Bonferroni tests. A probability of *p* < 0.05 was accepted as statistically significant. The summary data are presented as means ± SEM where n refers to the number of vessels in an experimental group with 1 PCA studied per mouse per preparation. Findings are from male and female C57BL/6J and PAI-KO mice.

## Data Availability

Data supporting the findings of this study are located at https://doi.org/10.7910/DVN/BZXNI0 (accessed on 31 July 2024).
